# Accuracy of the Automated Range of Motion Observer and Reporter Software for Fully Automated Joint Measurement From Patient Videos

**DOI:** 10.5435/JAAOSGlobal-D-25-00088

**Published:** 2025-07-08

**Authors:** Sundeep Chakladar, Christopher J. Dy, David M. Brogan

**Affiliations:** From the Department of Orthopaedic Surgery (Chakladar), Washington University School of Medicine in St. Louis, St. Louis, MO, and the Division of Hand and Microsurgery (Dr. Dy, Dr. Brogan), Department of Orthopaedic Surgery, Washington University School of Medicine in St. Louis, St. Louis, MO.

## Abstract

**Introduction:**

Accurate assessment of joint range of motion (ROM) is essential for diagnosing and managing upper extremity injuries. Universal goniometers are the most used tools for measuring ROM, but they require skilled observers and are limited by interobserver variability. An automated system for measuring joint range of motion from patient videos could facilitate standardized reporting of outcomes after reconstructive surgery.

**Methods:**

An Automated Range of Motion Observer and Reporter (ARMOR) software was developed as an autonomous, video-based ROM measurement tool leveraging OpenCV pose estimation. ARMOR was used to assess upper extremity range of motion and was validated against photography-based (photogoniometry) and manual goniometry in a cohort of brachial plexus surgery patients.

**Results:**

The correlation coefficients comparing ARMOR to manual goniometry were above 0.90 for all motion tasks, except for elbow flexion. For shoulder flexion, the mean difference between ARMOR and manual goniometry was more than 14° smaller than the difference for photogoniometry. Mean differences for shoulder abduction were similar between ARMOR and photogoniometry.

**Conclusion:**

ARMOR's accuracy in assessing shoulder ROM, independence from human observer bias, and telemedicine compatibility makes it a promising solution for consistent and accessible ROM assessment. The autonomous nature of the software enhances the data collection workflow for clinical researchers while eliminating interrater variability.

Accurate measurement of joint range of motion (ROM) is an integral component of diagnosis, treatment, and postsurgical care in patients with upper extremity injuries. The universal goniometer is the most widespread tool used to assess joint mobility.^1^ Its low cost, portability, and ease of use have contributed to it becoming the benchmark within clinical settings.^2,3^ Goniometers can provide an accurate assessment of ROM when used correctly, but the quality of measurements is highly dependent on the experience of the tester and the specific techniques used.^4-7^

Identification of manual goniometry's poor interobserver reliability has prompted several researchers to develop digital tools that increase the accuracy, reliability, and clinical efficiency of upper extremity ROM measurement.^8^ One of the most widespread technologies that has been adapted for clinical goniometry is Microsoft's Kinect motion-sensing device.^8^ Huber et al^9^ reported high reliability and moderate accuracy of a Kinect-based system when measuring shoulder joint angles in healthy participants. Zulkarnain et al^10^ further demonstrated high accuracy and reliability when using Kinect to measure shoulder angles in both static and live models. Despite its analytical validity, widespread use of Kinect as a ROM measurement tool is limited by the cost, accessibility, and portability of its equipment.

Recent expansions in telemedicine have prompted interest in developing a fully virtual ROM measurement tool. Unlike Kinect, which requires specialized equipment, new goniometry technologies are leveraging the power of readily accessible devices.^8^ Several photography-based technologies fall into this category, and they each follow a similar methodological schema: a picture is obtained of an individual performing a range of motion task; this picture is then sent to a clinician, who uses digital tools to annotate and calculate joint angles. The pictures can be obtained through digital cameras, which serve as a secure method for clinicians to collect patient data, or through smartphone cameras, which are convenient for patients to use at home to collect their own images. The software used by clinicians for analysis of these images vary, but representative examples include ImageJ and the GNU Image Manipulation Platform (GIMP), which have built-in angle measurement tools. As described by Zhao et al,^11^ when using these tools, the clinician first manually identifies three reference points for a given joint. The program then automatically calculates the angle formed by these points. These reference points include the joint of interest and the two large joints directly connected to it. For example, when calculating right elbow flexion, the reference points would be the right elbow, right wrist, and right shoulder. This method has been termed “photogoniometry” due to its assessment of joint angles through photography.^12^

Zhao et al^11^ evaluated the accuracy of GIMP-based photogoniometry and found that it closely matched the results of manual goniometry, with a mean difference of 3.0 to 3.2°. Repeated values obtained by the same rater using the photogoniometric method had Pearson correlation coefficients ranging from 0.92 to 0.94, indicating high reliability. Several researchers have done similar evaluations of photogoniometry in relation to manual goniometry, with results consistently displaying moderate to high accuracy and reliability.^12-14^ The data collection step of photogoniometry is suitable for telehealth, as participants can easily use a tripod and smartphone to obtain images of themselves performing range of motion tasks at home. However, this method requires image interpretation by a clinician, which decreases efficiency and introduces opportunities for interobserver variation in reported measurements.

To address these limitations, Quang et al incorporated the use of a pose estimation algorithm into the process of image analysis. Rather than a clinician manually annotating images, pose estimation algorithms use machine learning models to automatically detect the location of major joints, like the shoulder, elbow, wrist, and hips. Once these reference points are identified, the joint angle of interest can be obtained by the observer inputting their coordinates into a simple geometric formula. Quang et al^15^ found their pose estimation model to be highly accurate in comparison to manual goniometry, with mean differences ranging from 0.9 to 2.0°. Similar experiments have been done using other pose estimation algorithms, including MediaPipe-Hands, HybridPoseNet, and AlphaPose, with their results confirming high accuracy and reliability.^16-18^

Despite favorable findings, these approaches have a notable limitation. When an individual performs a ROM task, an observer must identify the moment of maximum ROM, which the pose estimation models cannot do. Two approaches have been used to address this: either the participant self-identifies the maximum ROM or an observing clinician does.^15-18^ Both methods are less than ideal, as they can lead to inefficiency, errors, and interobserver variation.

Despite rapid development of digital ROM measurement technologies, there is still a need for a fully autonomous tool that provides consistent measurements across all observers and integrates easily into telemedicine. Therefore, we aimed to design a tool that uses a pose estimation model to measure joint angles, while also identifying the frame of a video in which an individual achieves maximal ROM. This resulted in the development of the Automated Range of Motion Observer and Reporter (ARMOR), which uses the OpenCV pose estimation algorithm to identify the location of an individual's joints in each frame of a provided video. The coordinates of these joints are used to calculate the angle of a specified joint. The accuracy of the OpenCV algorithm in the context of upper extremity goniometry has not been studied. However, the demonstrated success of similar pose estimation algorithms in ROM measurement indicates its potential effectiveness.

Most studies evaluating new goniometry tools have used samples consisting solely of healthy individuals.^8^ Therefore, we aimed to evaluate the validity of ARMOR in the context of brachial plexus surgery patients, a population that often experiences functional loss of upper extremity joints. This study assessed the accuracy of ARMOR by comparing its measurements with those of photogoniometry and manual goniometry.

## Methods

### Participants

Eight patients (all males, with an average age of 47 ± 18 years) with a history of brachial plexus injury and associated reconstruction surgery were selected as participants in this study. Average time since reconstruction was 21 ± 18 (range, 3–62) months. Cause of injury was traumatic in six patients and iatrogenic in two patients. Five patients had upper trunk or upper trunk extended injuries; one had a panplexus injury; and two had lower trunk injuries.

Each participant did three different range of motion tasks: shoulder abduction, shoulder forward flexion, and elbow flexion, using their injured limb. Written consent was obtained for use of their videos for research purposes.

### Video Setup

Participant distance from camera and height of camera were standardized in the measurement environment used in the study. Three digital cameras (Oiadek B0CGQX3HB9) were used in the environment—one directly in front and one on either side of the participant—with each being six feet from the participant. The cameras were set at a height of five feet, approximately level with the participants' shoulders.

### Recording Motion Tasks

Participants were instructed on how to perform each motion task by a member of the research team, who was also present while the tasks were done to ensure that directions were followed. The anterior view camera was used to obtain shoulder abduction videos. The lateral view camera that corresponded to the side of the participant's injured limb was used for obtaining shoulder forward flexion and elbow flexion videos. Because ARMOR only tracks motion in a two-dimensional plane, participants were instructed to only move their joints in a plane parallel to the camera's view. In addition, participants were asked to minimize elbow displacement during elbow flexion and to minimize shoulder displacement during shoulder abduction and flexion.

### Range of Motion Measurement by ARMOR

**Figure 1:**
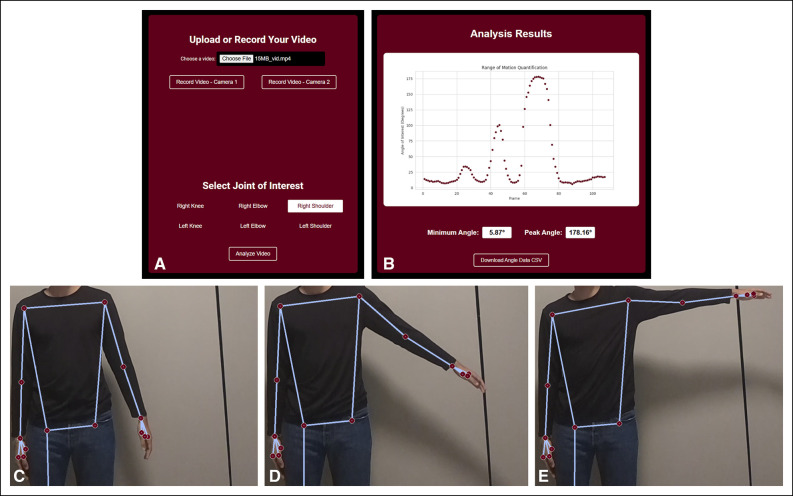
ARMOR Web application interface and visualization of pose estimation analysis. **A,** ARMOR web application upload page where users upload or record a motion task video, select a joint to measure, and submit the video for analysis. **B,** Results page displaying ARMOR's analysis output as a scatterplot, with an option to download the data as a .csv file. **C**–**E,** Sequence of images showing pose estimation analysis at different timepoints from a sample video of an individual performing left shoulder abduction.

The Python code for ARMOR was adapted into a web app (armor-app.onrender.com) to streamline data upload and result retrieval (Figure 1, A and B). Figure 1, C–E show the software performing pose estimation analysis at different timepoints on a sample uploaded video. The locations of primary joints (wrists, elbows, shoulders, hips, knees, and ankles) were approximated by the OpenCV pose estimation library (version four.x). ARMOR then calculated the angles of shoulder abduction and shoulder forward flexion using the following formula:

$$|\;\frac{\pi }{180}\;\mathrm{atan}\;2\left(BA\times BC,BA\cdot BC\right)\;|,$$

with BA corresponding to the vector made by the shoulder joint and the elbow joint (shoulder-elbow vector) and BC corresponding to the shoulder-hip vector. The angle for elbow flexion was calculated using the following formula:

$$180-\left|\;\frac{\pi }{180}\;\mathrm{atan}\;2\left(BA\times BC,BA\cdot BC\right)\;\right|,$$

with BA corresponding to the elbow-wrist vector and BC corresponding to the elbow-shoulder vector. Joint angles were calculated for each frame of the motion task video. The largest calculated angle across all frames was identified as the participant's maximum ROM for that task.

### Range of Motion Measurement by Photogoniometry

Eight physicians with prior goniometry experience were provided with the same videos submitted to ARMOR. Their method of assessing joint ROM closely aligned with the photogoniometry methods described by Smith et al. and Meals et al. For each video, the physician first identified the frame in which they believed that the participant achieved maximum abduction/flexion. They then used ImageJ (Fiji) version 1.54j to annotate the location of three points that corresponded to joint of interest and the two large joints directly connected to it. For shoulder abduction and forward flexion, these reference points were the shoulder, wrist, and hip of the side being assessed. For elbow flexion, reference points were the elbow, wrist, and shoulder. The Angle Tool in ImageJ was used to calculate the angle formed by these three points, with the vertex at the joint of interest. This angle was determined to be the maximum ROM for the participant for that specific motion task. Physicians had access to all three video views (anterior and bilateral) for better joint visualization but only made annotations on the view used by ARMOR for each motion task.

Physicians performed these measurements independently of each other, without knowledge of the output provided by ARMOR. For each video, results across physicians were averaged to obtain a single consensus ROM value (photogoniometry mean).

### Range of Motion Measurement by Manual Goniometry

A separate physician, who was not involved in photogoniometric assessment, manually measured the ROM of participants during each of the motion tasks using a universal goniometer. These measurements were obtained during the same session that the motion task videos were recorded.

### Statistical Analysis

To compare the results of ARMOR and photogoniometry, we have done a linear regression analysis on their output data for each of the three motion tasks. An R-square value was calculated to determine their degree of correlation. The Student *t* test was also used for each motion task, with *P* values less than 0.05 indicating a notable difference between the outputs of the two measurement methods.

The accuracy of ARMOR and photogoniometry were both assessed by comparing their outputs with those of manual goniometry, which has been established as the benchmark for clinical measurement of joint ROM. This first involved calculating the mean difference between ARMOR/photogoniometry and manual goniometry for each motion task. Strength of correlation between the digital methods and manual goniometry was then assessed by interclass correlation coefficient (ICC) and Pearson correlation coefficient (Pearson-CC). Following the cutoffs used by Gu et al., ICC values were interpreted as follows: <0.20: unacceptable, 0.20 to 0.40: questionable, 0.41 to 0.60: good, 0.61 to 0.80 very good, >0.81: excellent. For Pearson-CC values, one indicates a total positive linear correlation, 0 means no linear correlation, and −1 shows a total negative linear correlation.

For each motion task, interobserver reliability of photogoniometry was assessed by calculating the intraclass correlation coefficient and the average coefficient of variation across all patients. Intraclass correlation coefficients were interpreted using the same cutoffs described above for ICC values. Average coefficients of variation were interpreted as follows: <10%: low variability, 10 to 20% moderate variability, and 20%: high variability.

Assessing the interobserver reliability of photogoniometry was necessary due to potential variations between observers. This variation arises because the procedure involves manual selection of the appropriate video frame and manual annotation of reference points. Consequently, even when the eight physician raters are given the same video, they may produce different results. By contrast, ARMOR eliminates any variation between observers because the video analysis procedure does not involve manual interpretation. It uses equations to automatically identify joint positions and the appropriate video frame, ensuring identical results each time the same video is analyzed. As it is assumed to have perfect interrater reliability, the reliability of ARMOR was not explicitly assessed.

All statistical analyses were done in R version 4.3.2. The icc() function from the irr package was used to calculate intraclass and interclass correlation coefficients, employing the two-way random-effects model for assessing reliability.

## Results

### Agreement Between ARMOR and Photogoniometry

**Figure 2:**
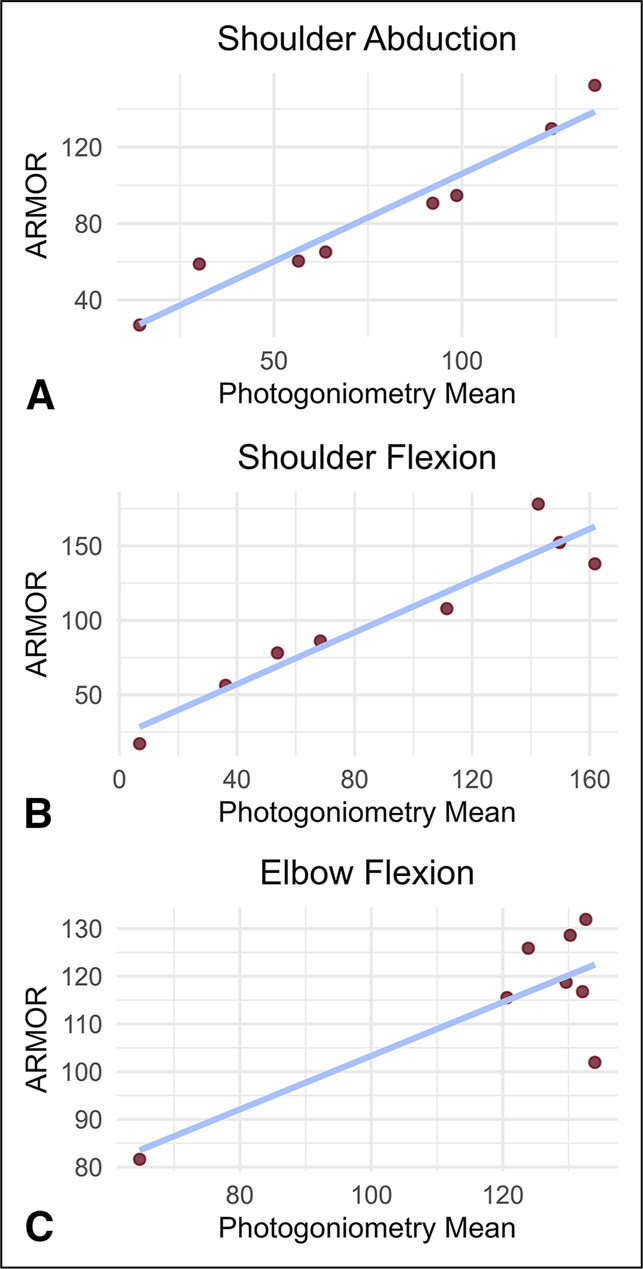
Linear regression analysis of photogoniometry and ARMOR. **A,** Shoulder abduction, R-square = 0.9366. **B,** Shoulder flexion, R-square = 0.8987. **C,** Elbow flexion, R-square = 0.6281. Each point represents an individual video. All values are measured in degrees.

Linear regression analysis revealed positive correlations between photogoniometry and ARMOR across all tasks, with R-square values ranging from 0.6281 to 0.9366 (Figure 2, A–C). For the assessment of shoulder abduction, the Student t test revealed no notable statistical difference between the two methods (*P* = 0.7336). By contrast, the test found notable differences between the outputs for shoulder flexion (*P* = 0.0379) and elbow flexion (*P* = 0.0392).

### Accuracy of ARMOR and Photogoniometry compared with Manual Goniometry

Table 1 presents the results of the statistical comparison between the digital goniometry methods and manual goniometry. All comparisons showed excellent interclass correlation coefficients, except for the comparison between ARMOR's and manual goniometry's elbow flexion outputs, which indicated a good correlation. Pearson-CCs showed a similar trend, with all comparisons exceeding 0.9, except for the elbow flexion comparison between ARMOR and manual goniometry, which had a value of 0.824. Mean differences were similar for shoulder abduction. For shoulder flexion, ARMOR had a mean difference approximately 14° lower than photogoniometry compared with manual goniometry. This trend was reversed for elbow flexion, as ARMOR's mean difference was approximately 10° higher.

**Table 1:** Comparison Digital Goniometry Outputs to Manual Goniometry

		Mean Difference (^o^)	Pearson-CC	ICC
Shoulder abduction	ARMOR vs. MG	13.663	0.955	0.925
	Photogoniometry vs. MG	12.281	0.942	0.929
Shoulder flexion	ARMOR vs. MG	8.323	0.983	0.979
	Photogoniometry vs. MG	22.375	0.903	0.910
Elbow flexion	ARMOR vs. MG	24.651	0.824	0.787
	Photogoniometry vs. MG	14.578	0.980	0.840

MG = manual goniometry, Pearson-CC = Pearson correlation coefficient, ICC = interclass correlation coefficient.

### Reliability of Photogoniometry

Intraclass correlation coefficients for photogoniometry measurements were as follows: shoulder abduction = 0.959, shoulder flexion = 0.981, and elbow flexion = 0.956. Average coefficient of variation across patients for these measurements were as follows: shoulder abduction = 14.970%, shoulder flexion = 11.416%, and elbow flexion = 4.097%.

## Discussion

Accurate and reliable ROM measurement is important for the management and treatment of upper extremity injuries. Universal goniometers have become the benchmark for this measurement, but their variable interobserver reliability has prompted the search for alternative digital tools.^4-7^ Photogoniometry has emerged as an inexpensive, easily implemented tool for ROM measurement, and several studies have validated its accuracy and reliability.^11-14^ However, photogoniometry requires interpretation by a clinician, which decreases efficiency and introduces opportunities for interrater variability. Given these limitations, pose estimation algorithms serve as a promising alternative, as they can automatically identify joint locations in an image, removing the need for manual annotation of joint positions. Although open-source pose estimation algorithms can produce accurate and reliable ROM measurements, current protocols still require manual identification of the moment an individual achieves maximal ROM, which may introduce interobserver variability.^3^

This study aimed to develop and validate a fully autonomous, video-based, ROM measurement tool that delivers reliable results and can be easily integrated into telemedicine. We specifically focused on its efficacy in individuals who had recently undergone brachial plexus surgery, as most studies that assess new goniometric tools have only included healthy, asymptomatic participants.^8^

We compared the results of ARMOR and photogoniometry to determine if notable differences were found in their measurements of shoulder abduction, shoulder flexion, and elbow flexion. Linear regression analysis showed strong positive correlations between the two methods for shoulder ROM assessments, with a moderately positive correlation for elbow flexion. This suggests consistency between the methods, especially for shoulder abduction, which had an R square value of 0.9366. Along with regression analysis, we used the Student *t* test to check for statistically significant differences between the two methods' outputs. We found no notable difference in shoulder abduction measurements. However, the results for shoulder flexion and elbow flexion both had *P*-values below 0.05, indicating notable differences between the methods. These findings prompted the need to identify whether ARMOR or photogoniometry were more accurate in their assessment of shoulder and elbow flexion.

The accuracies of ARMOR and photogoniometry were assessed by comparing their results across all three motion tasks with those of manual goniometry. Pearson-CCs exceeded 0.9 for all comparisons, except for the elbow flexion comparison between ARMOR and manual goniometry, which still had a moderately high value of 0.824. This demonstrates a strong correlation between the digital and manual methods. ICCs for shoulder abduction were equally high for both ARMOR and photogoniometry, indicating equally excellent agreement with manual goniometry. Both ICCs for shoulder flexion were also above 0.9. However, the difference between these values was much larger than that of shoulder abduction, indicating that ARMOR's shoulder flexion outputs have moderately higher agreement with manual goniometry. The ICCs for elbow flexion were appreciably lower than the previous motion assessments, especially for ARMOR, which was the only comparison to not demonstrate excellent agreement with manual goniometry.

The third method of assessing accuracy of ARMOR and photogoniometry was mean difference between their outputs and manual goniometry. Mean differences differed less than 2° for shoulder abduction, further confirming similar levels of accuracy between ARMOR and photogoniometry for this motion task. ARMOR's mean difference for shoulder flexion was more than 14° less than that of photogoniometry, indicating that ARMOR is markedly more accurate for the assessment of shoulder flexion. The inverse is true for elbow flexion, as photogoniometry had a mean difference of more than 10° smaller than that of ARMOR.

The analyses thus far identify that for shoulder abduction, ARMOR and photogoniometry produce statistically similar results that are equally accurate. The same is not true for shoulder and elbow flexion, as the two methods produce results that are markedly different. Comparison to manual goniometry reveals that ARMOR is more accurate than photogoniometry for shoulder flexion, whereas photogoniometry is more accurate for elbow flexion.

We suspect ARMOR excelled in assessing shoulder flexion because compared with human annotation, it more accurately identifies shoulder location. In the lateral view videos of forward flexion, the shoulder often became less visible due to bulky clothing like dress shirts, vests, and winter coats. This decreased visibility likely prevented the physician observers from accurately identifying the location of the shoulder joint. This issue did not appear in anterior view videos of shoulder abduction, likely because surrounding anatomy and background contrast allowed for reliable identification of the shoulder. ARMOR can accurately identify shoulder location even when it may be obstructed by clothing because it does not solely rely on shoulder visibility. Instead, it uses the location of surrounding joints—like the elbow, hip, and opposite shoulder—to interpret the true location of the shoulder.

We suspect that ARMOR's inaccuracy in assessing elbow flexion is due to the nature of the motion task. ARMOR's algorithm assumes that all joint movements occur in a two-dimensional plane, but the task of touching the hand to the mouth—as participants were instructed to do—requires some three-dimensional motion, which ARMOR does not account for. Physicians performing photogoniometry can easily adjust for this three-dimensional movement by adjusting the placement of their reference points, but ARMOR's inability to do so may have led to skewed results.

After evaluating the accuracy of the digital goniometry methods, we aimed to assess the reliability of photogoniometry. The intraclass correlation coefficient for photogoniometry exceeded 0.9 for all three motion tasks, indicating excellent reliability. Coefficients of variation showed low variability for shoulder abduction and moderate variability for shoulder and elbow flexion. The high intraclass correlation coefficients for these flexion tasks suggest that most of the variability is due to actual differences between participants rather than inconsistencies in observer measurements.

Photogoniometry shows high reliability, but some variation still exists between observers' measurements. This variation is either due to differences in how physicians selected reference points or identified the frame representing the moment of maximal ROM. By contrast, ARMOR performs these processes using an algorithmic approach, ensuring that for a given video, the same frame and same reference points are always selected.

Our novel application of the OpenCV pose estimation algorithm shows variable accuracy when compared with photogoniometry, which has consistently been implicated as a valid alternative to manual goniometry.^19-21^ Our results suggest that ARMOR is better equipped than photogoniometry for the assessment of shoulder flexion. Conversely, photogoniometry displayed higher accuracy than ARMOR for the measurement of elbow flexion. Similar levels of accuracy in their assessment of shoulder abduction indicate that ARMOR and photogoniometry can be used interchangeably for this motion task.

An advantage of our method is that it mitigates the prevalence of human bias or error. Frame selection, joint localization, and angle calculation are done by computational algorithms, which ensures consistent, reliable results once the video has been obtained. In addition, patients can easily record and submit videos to ARMOR from their own homes. Our experimental setup involved three cameras positioned around the patient. Physicians used all three views for photogoniometry, whereas ARMOR only required one view for each of its analyses. Implementation of our model only requires a smartphone to record and upload motion task videos to the ARMOR web app and a tripod to stably position the camera.

A notable limitation of this study is its sample size. We did a power analysis, setting the values of the mean of paired differences as 5°, the standard deviation of paired differences as 9°, alpha as 0.05, and power as 0.85. This resulted in a sample size of 30, which closely aligns with the average sample size in studies evaluating digital goniometric tools.^8^ Because of the low incidence of brachial plexus injuries, we were limited to eight participants. Assessing ROM in these patients is challenging due to their muscle and nerve dysfunction, leading us to explore whether our new tool was applicable in this complex patient population. Given that the inclusion of healthy patients may have skewed the results, we chose to not include them and instead accept a smaller sample size. Future studies of this technology can use a multi-institutional approach to attain a sufficient sample.

## Conclusion

This study introduces a reliable and accessible method for video-based, upper extremity ROM measurement. Accuracy of the technology in assessing shoulder abduction and forward flexion was confirmed through comparisons with photogoniometry and manual goniometry. Our results implicated this algorithmic approach as a novel, alternative approach to manual goniometry. This technology has notable potential for integration into telemedicine because patients can measure their own joint mobility through self-obtained videos, eliminating the need for in-clinic appointments with skilled members of the healthcare team. The technology was found to have low accuracy in its assessment of elbow flexion, which encourages further improvement and exploration of pose estimation models that are better able to account for three-dimensional joint motion. Additional studies involving larger patient populations are recommended to validate the technology's effectiveness across various upper extremity injuries and expand its applicability in broader clinical settings.
